# Case report: Therapy adherence, *MTTP* variants, and course of atheroma in two patients with HoFH on low-dose, long-term lomitapide therapy

**DOI:** 10.3389/fgene.2022.1087089

**Published:** 2023-01-04

**Authors:** Meral Kayikcioglu, Hasan Selcuk Ozkan, Burcu Yagmur, Selen Bayraktaroglu, Asli Tetik Vardarli

**Affiliations:** ^1^ Department of Cardiology, Ege University School of Medicine, Izmir, Turkey; ^2^ Ege University School of Medicine, Izmir, Turkey; ^3^ Department of Radiology, Ege University School of Medicine, Izmir, Turkey; ^4^ Department of Medical Biology, Ege University School of Medicine, Izmir, Turkey

**Keywords:** MTTP, lomitapide, homozygous familial hypercholesterolemia, lipoprotein apheresis, low-density lipoprotein receptor, genetics

## Abstract

**Background:** Homozygous familial hypercholesterolemia (HoFH) is a rare and devastating genetic condition characterized by extremely elevated levels of low-density lipoprotein cholesterol (LDL-C) leading to an increased risk of premature atherosclerosis. Patients with Homozygous familial hypercholesterolemia mostly present with mutations in *LDLR*; however, herein, we present two cases with concomitant *microsomal triglyceride transfer protein* mutations, who showed different clinical courses and treatment adherence on long-term therapy with the new MTTP inhibitor lomitapide.

**Objectives:** We aimed to present the possibility of preventing the progression of atherosclerotic burden with effective and safe LDL-C reduction in patients with Homozygous familial hypercholesterolemia on low-dose lomitapide therapy and emphasize the role of treatment adherence in therapy success.

**Methods:** We present two patients with phenotypically Homozygous familial hypercholesterolemia, a compound heterozygous woman and a simple homozygous man, both with *LDLR* and additional *MTTP* mutations, who were treated with the MTTP-inhibiting agent lomitapide, with different treatment compliances. The role of impulsivity was investigated through *Barratt Impulsivity Scale 11*, and the extent of the atherosclerotic burden was followed up using coronary artery calcium scoring, echocardiographic and sonographic findings, and, eventually, through a strict follow-up of laboratory parameters. The patients were on lomitapide for 8 and 5 years, respectively, with no adverse effects.

**Conclusion:** When accompanied by good adherence to therapy, low-dose lomitapide on top of standard lipid-lowering therapy with decreased frequency of lipid apheresis prevented the progression of atherosclerotic burden. Non-compliance might occur due to patient impulsivity and non-adherence to a low-fat diet.

## 1 Introduction

Homozygous familial hypercholesterolemia (HoFH) is a rare metabolic disorder of mainly autosomal-dominant inheritance that causes extremely high low-density lipoprotein (LDL) cholesterol levels (Rosenson, 2021). Affected individuals show elevated LDL cholesterol levels from birth, which leads to premature atherosclerotic cardiovascular disease (ASCVD). Therefore, the early detection of this condition is critical to prevent early morbidity and mortality (Kayikcioglu et al., 2014; Zhang et al., 2020; Tokgozoglu and Kayikcioglu, 2021). At least four genes are associated with familial hypercholesterolemia (FH), including *LDLR*, *APOB*, *PCSK9*, and *LDLRAP1*, with phenotypic variations (Prince et al., 2013; Chemello et al., 2021; Tokgozoglu and Kayikcioglu, 2021). In most cases, the underlying mutation is in *LDLR*, which is associated with typical LDL cholesterol levels >500 mg/dl in patients with HoFH. Due to absent or defective LDL-receptor activity, individuals with HoFH are resistant to conventional lipid-lowering therapy (LLT) targeting LDL-cholesterol clearance by upregulating LDL receptors. Therefore, LDL apheresis is the most effective treatment for these patients (Kayikcioglu et al., 2018). However, the semi-invasive and time-consuming nature of apheresis is the major obstacle leading to decreased quality of life, increased risk of depression, and deterioration in mental status, leading to high treatment refusal and low adherence (Kayikcioglu et al., 2019; Tunçel et al., 2020). Therefore, effective pharmacotherapies with long-term safety are needed for patients with HoFH.

Lomitapide is a microsomal triglyceride transfer protein (MTTP) inhibitor that reduces LDL cholesterol levels through LDL receptor-independent pathways by directly decreasing apoprotein (Apo)-B levels (Cuchel et al., 2013). Herein, we present two patients with HoFH on long-term, low-dose lomitapide without drug adverse effects but different atheroma courses despite similar baseline LDL cholesterol levels.

## 2 Case descriptions

**FIGURE 1:**
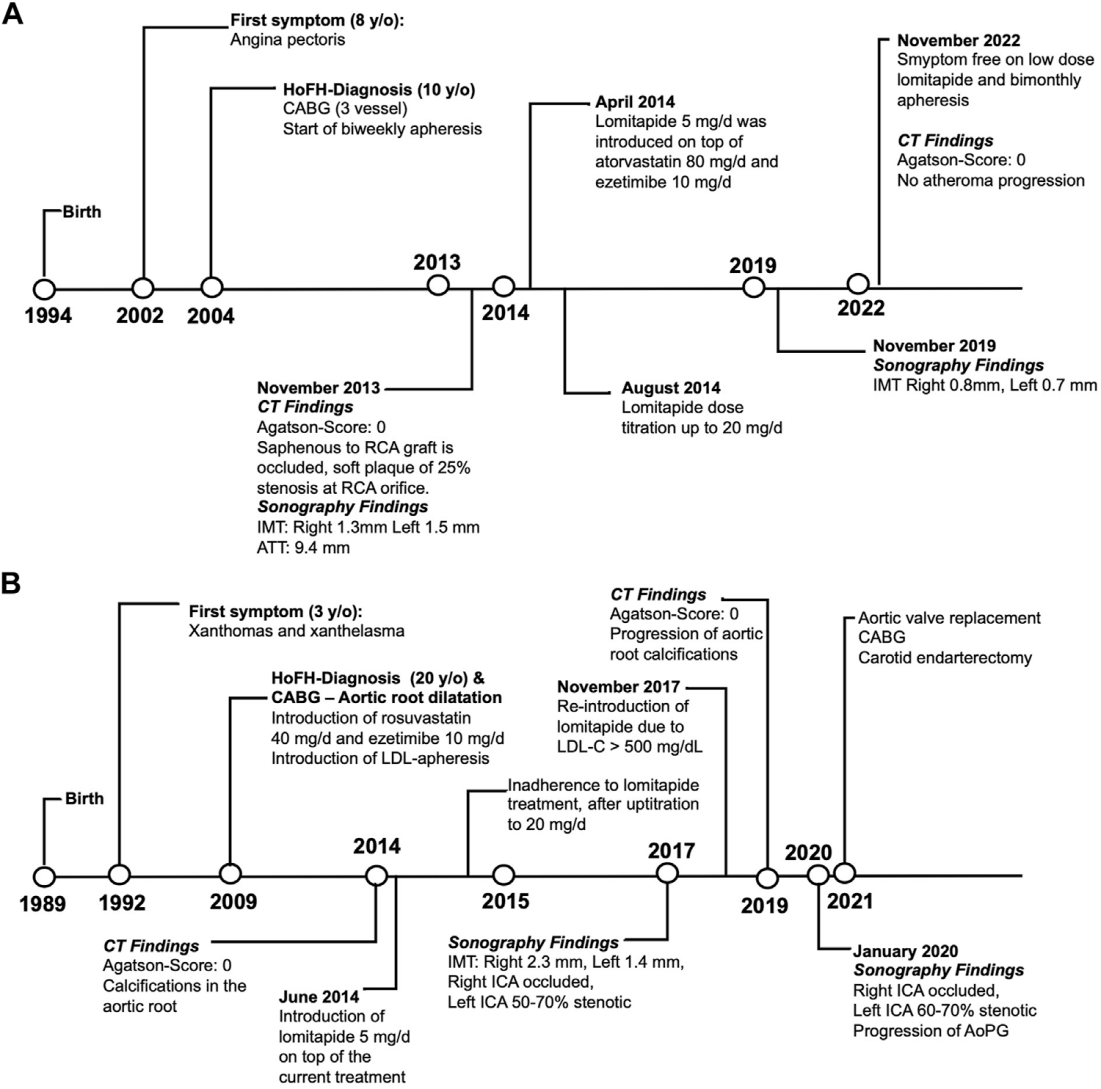
Timeline schema representing the clinical evolutions of Cases 1 **(A)** and 2 **(B)**. Abbreviations*:* CABG, coronary artery bypass graft; CT, computed tomography; ICA, internal carotid artery; RCA, right coronary artery; IMT, intima-media thickness; ATT, Achilles tendon thickness; HoFH, homozygous familial hypercholesterolemia; AoPG, aortic peak gradient; LDL-C, low-density lipoprotein cholesterol.

Both patients were undergoing long-term follow-up at the Ege University Medical School, Department of Cardiology, Lipid Clinic. They were selected by the means of need and after receiving consent for the compassionate use of lomitapide as an add-on therapy. The clinical characteristics of the patients before and after lomitapide therapy are shown in Table 1. Figure 1A,B depicts the timeline of the case symptoms, diagnosis, and management.

**TABLE 1:** Clinical characteristics of the cases.

	Case-1	Case-2
Gender/age (years)	Female/28	Male/33
Age at initial symptom (years)	8, angina	3, xanthomas
Age at diagnosis (years)	10	20
Age of onset of CV disease (years)	8	20
Age at first LDL-apheresis (years)	10	22
Age at the introduction of lomitapide therapy (years)	20	24
CV disease	CABG in 2004	CABG in 2009
	(at age 10 years)	(at age 20 years)
	No history of MI	No history of MI
Weight (kg)	55	88
Height (cm)	160	172
Body mass index (kg/m^2^)	21.5	29.7
Blood pressure (mmHg)	98/62	118/78
Hypertension	None	None
Smoking sfttatus (pack-years)	None	None
Diabetes	None	None
Consanguinity of parents (degree)	Second-degree	Second-degree
Family history of CV disease	Remarkable for premature MI both in maternal and paternal sides	Remarkable for premature MI both in maternal and paternal sides
	Father had a mortal CV event at age 38	
Xanthomas	At diagnosis she had on her knees but none now	At diagnosis, he has extensive xanthomas on his knees, elbows, fingers, buttocks, *etc.* Now, most of them vanished but still some on his knees
Corneal arcus	None	Yes
Genetic analysis		
- *LDL-R*	Heterozygous	Homozygous
	c.664T>C, (p.Cys222Arg)	c.1760dupG, (p.Ser587ArgfsTer16)
- *Apo-B*	-	-
- *PCSK9*	-	-
- *LDLRP1*	-	-
- *MTTP*	rs3816873, rs1061271, rs11944749, rs17029189, rs11944752, rs17029213, rs17029215, rs2306985, rs2718684 rs34734558, rs41275719, rs7667001, rs881981, rs982424, and rs991811	rs3816873 and rs1061271
Imaging characteristics		
A. Carotid ultrasonography		
- At baseline before lomitapide	IMT Right 1.3 mm and center 1.5 mm	IMT Right 2.3 mm
	Right ICA minor soft plaque	Left 1.4 mm
	(November 2013)	Right ICA is occluded
		Left ICA 50–70% stenotic calcific plaques
		(2017)
- On lomitapide	IMT Right 1.0 mm and Left 1.0 mm	-
	Right ICA minor calcific plaque	
	(December 2014)	
	IMT Right 0.8 mm and center 0.7 mm	-
	Right ICA minor calcific plaque	
	(November 2015)	
	IMT Right 0.8 mm and center 0.7 mm	Right ICA is occluded
	Right ICA minor calcific plaque	Left ICA 60–70% stenotic calcific plaques
	(November 2019)-	(January 2020)
B. Achilles thickness (mm)- Ultrasound		
- At baseline before lomitapide	9.4 mm	9.8 mm
	(November 2013)	
- On lomitapide	7 mm	NA
	(December 2014)	
	4.8 mm	NA
	(November 2019)	
C. Coronary artery calcium score (Agatston units)	Zero (in all CT evaluations)	Zero (in all CT evaluations)
D. Coronary computerized tomography		
- Before lomitapide	Date: 2013	Date 2014
	Both the bypass grafts of LIMA to LAD and saphenous obtuse marginal graft are patent. The saphenous to RCA graft is occluded. A soft plaque of 25% stenosis at RCA orifice	There are soft plaques and ostial stenosis at the level
		of proximal RCA and LMCA. Atherosclerotic calcifications are present in the aortic root and ascending aorta
- On lomitapide	Date: 2022	Date 2019
		There are soft plaques and ostial stenosis at the level
	Both the LIMA graft to LAD and saphenous obtuse marginal graft are patent. The saphenous to RCA bypass graft is occluded. A soft plaque of 25% stenosis at RCA orifice	of proximal RCA and LMCA
		There are focal stenosis and wall thickening at the level of the saphenous vein graft placed to
		obtuse marginal branch. Atherosclerotic calcifications are present in the aortic root and ascending aorta (progressed compared to previous CT)
E. Echocardiographic features		
-Before lomitapide	Date: 2013	Date: 2015
	Degenerated aortic cusps	Septal-lateral hypokinesia
	Mild aortic regurgitation	Sclerotic aortic valves
	Mild-to-moderate aortic stenosis	Mild aortic regurgitation
	Mean gradient: 23.00 mmHg	Mean gradient: 11.00 mmHg
	Peak gradient: 39 mmHg	Max gradient: 25.00 mmHg
		Minimal mitral regurgitation
		Minimal tricuspid regurgitation
- On lomitapide	Date: 2021	Date: 2018
	Degenerated aortic cusps	Degenerated calcific aortic valve
	Aortic regurgitation: Mild	AVA: 1.12 cm^2^
	Mild-to-moderate aortic stenosis	Mild aortic regurgitation
	Mean gradient: 26 mmHg	Moderate aortic stenosis
	Peak gradient: 41 mmHg	Mean gradient: 29 mmHg
		Peak gradient: 47 mmHg
		Date: 2019
		Moderate aortic stenosis
		Mean gradient: 35 mmHg
		Peak gradient: 60 mmHg
		Date: 2020
		Moderate aortic stenosis
		Mean gradient: 36 mmHg
		Peak gradient: 68 mmHg
		AVA: 1.00 cm^2^
Lipid and lipoproteins		
1. LDL-cholesterol (mg/dl)		
At diagnosis	519	582
On apheresis		
At the end of the first year of apheresis pre- and post-session (mean)	146–77	261–147
	Weekly and biweekly apheresis	Weekly and biweekly apheresis
At the end of the fifth year of apheresis pre- and post-session (mean)	249–90	466–276
	Every 2 weeks apheresis	Monthly apheresis
At the last three sessions on before lomitapide pre-post session (mean)	330–175	479–322
On lomitapide		
- Baseline	248	562
- 1st month–lomitapide 5 mg/day	170 (no apheresis)	413 (no apheresis)
- 6th month- lomitapide 20 mg/day	129 (no apheresis)	287
- 1st year pre- and post-apheresis session (3 sessions mean)	132–54	256–133
	Apheresis every 2 months	Apheresis every 2 months
2. Lipoprotein (a)		
- At diagnosis (mg/dl)	34.0	<7
- Before lomitapide (mean) (nmol/L)	28	<7
- After lomitapide (mean) (nmol/L)	21	<6
3. Lipid profile (mean, mg/dL) Before lomitapide		
- Total cholesterol	445	585
- Triglycerides	111	98
- HDL-C	32	31
- Apo A1	81	88
- Apo B	141	320
4. Lipid profile (mean, mg/dL) After lomitapide		
- Total cholesterol	198	352
- Triglycerides	78	70
- HDL-C	42	38
- Apo A1	96	99
- Apo B	79	224
Laboratory analysis (mean)		
AST/ALT (U/L) before lomitapide	24.50/27.40	16.17/15.6
AST/ALT (U/L) after lomitapide	17.92/17.14	13.00/10.00
Creatinine (mg/dl) before lomitapide	0.62	0.86
Creatinine (mg/dl) after lomitapide	0.73	0.87
Treatment		
** LLT at baseline before the start of lomitapide**	Atorvastatin 80 mg/day	Rosuvastatin 40 mg/day
	Ezetimibe 10 mg/day	Ezetimibe 10 mg/day
	LDL apheresis–every 15 days	LDL apheresis–monthly
** LLT (current)**	Atorvastatin 80 mg/day	Rosuvastatin 40 mg/day
	Ezetimibe 10 mg/day	Ezetimibe 10 mg/day
	Lomitapide 20 mg/day	Lomitapide 40 mg/day
	LDL apheresis (every 2 Months)	LDL apheresis (every 2 Months)
** Treatment duration on lomitapide**	8 years 1 month	5 years 9 months

CABG, coronary artery bypass grafting; LAD, left anterior descending; RCA, right coronary artery; LDL, low-density lipoprotein; HDL, high-density lipoprotein; HDL-C, high-density lipoprotein cholesterol; Apo, apolipoprotein; LIMA, left internal mammary artery; CV, cardiovascular; IMT, intima-media thickness; LLT, lipid-lowering therapy; AST/ALT, aspartate transaminase/alanine transaminase; CT, computed tomography; ICA, internal carotid artery.

### 2.1 Genetic analysis

Blood specimens were collected from the cases into tubes containing ethylenediaminetetraacetic acid. Genomic DNA was extracted from peripheral blood samples on a MagNA Pure LC instrument using the MagNA Pure LC DNA Isolation Kit I (Roche Applied Science, Germany). To amplify *LDLR*, *APOB*, *PCSK9, LDLRAP1,* and *MTTP* regions, the Ion AmpliSeq™ Designer tool was used to design specific assay primers to generate a custom FH-DNA panel based on the human (Hg19) reference genome (Ion AmpliSeq™ Target Technology, ThermoFisher Scientific, Waltham, MA). *LDLR*, *APOB*, *PCSK9, LDLRAP1,* and *MTTP* regions of the cases were sequenced on an Ion-Torrent PGM instrument (Life Technologies, Rockville, MD) (314-chip) according to the manufacturer’s recommended protocol. The Ion-Torrent suite 4.0 0.2 plugin was used to align the reads to the Hg19 reference genome. Integrative Genomics Viewer (IGV) v2.3 software http://www.broadinstitute.org/software/igv/download) was used to remove false-positive variations and possible PCR errors from the obtained variants. The Human Genetic Mutation Database (HGMD; http://www.hgmd.org/), the Variant Database (FHVD; http://www.ucl.ac.uk/ugi/fh), and https://varsome.com/databases were used for the annotation and prediction of the significance of the detected variants.

### 2.2 Case reports

Case 1 is a 28-year-old woman with HoFH receiving low-dose lomitapide combined with apheresis for 8 years. Her initial complaints started with chest pain at 8 years of age. She had undergone coronary artery bypass grafting (CABG) due to severe three-vessel coronary artery disease (CAD) at 10 years of age, when she was diagnosed with HoFH with an untreated LDL-cholesterol level ranging between 490 and 549 mg/dl. Her family history was remarkable for early-onset CAD and high cholesterol levels. She had received weekly or biweekly LDL-apheresis therapy since 10 years of age. As her LDL-cholesterol levels were far from the treatment goals, oral lomitapide 5 mg/dl was introduced on top of atorvastatin (80 mg/day) and ezetimibe (10 mg/day) at 20 years of age. The dose was titrated up to 20 mg/dl and has been maintained without adverse events. An additional 31% LDL-cholesterol reduction was observed in the first month of low-dose lomitapide (5 mg/day) therapy. At a dose of 20 mg/day, the LDL-cholesterol reduction reached 49% at the end of 6 months. As she declined to be weaned off apheresis, we reduced the apheresis frequency and she received low-dose lomitapide (20 mg/day) plus concomitant bimonthly apheresis. However, during the pandemic for 1 year, she could not access apheresis. Throughout the 8-year lomitapide treatment, she did not experience any increase in serum liver enzyme levels, weight loss, or liver steatosis. Moreover, no change was observed in hepatic fibro scans. She followed a fat-restricted diet compatible with lomitapide therapy to prevent steatorrhea, with concomitant supplementation of fat-soluble vitamins and essential fatty acids. Since the commencement of lomitapide therapy, the patient has remained stable at functional class (NYHA-I) without any adverse cardiovascular events.

**FIGURE 2:**
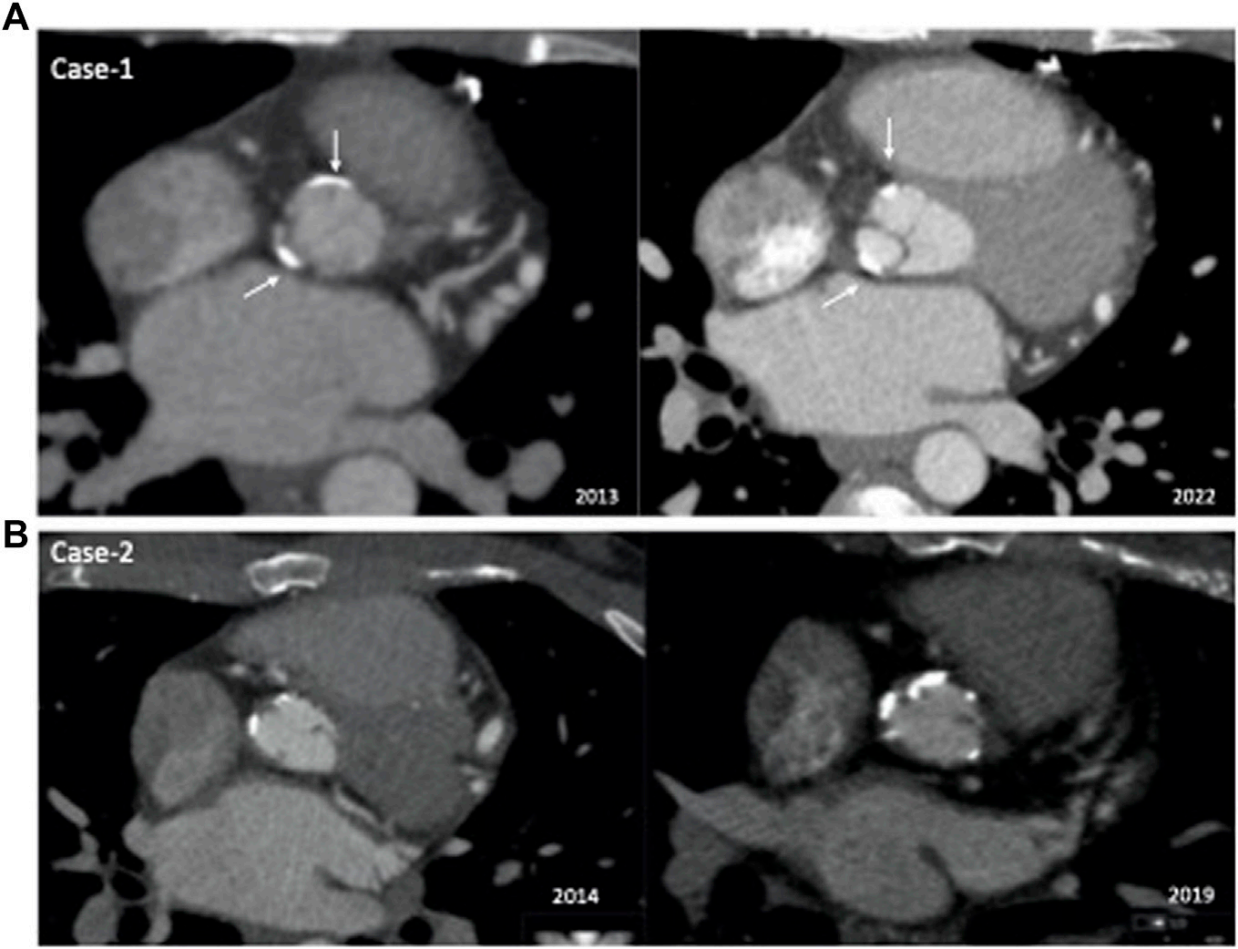
Comparison of baseline and follow-up computed tomography angiography images of the patients. **(A)** Case 1: Aortal involvement was almost identical between the baseline (2013) and follow-up (2022) images. **(B)** Case 2: Comparison of 2014 and 2019^−ΔΔCT^ angiograms showed significant progression of calcifications in the aortic root.

Imaging work-up at diagnosis revealed mild aortic stenosis [aortic peak gradient (AoPG) of 30 mmHg-mean (AoMG) 23–18 mmHg] and mild aortic regurgitation with a normal ejection fraction (EF). During the 8-year follow-up, the AoMG was stable at around 25–30 mmHg. In 2022, cardiac catheterization revealed an AoPG of 30 mmHg with normal EF. The computed tomography (CT) angiography showed almost identical coronary and aortal involvement between the baseline (2013) and follow-up (2022) images (Figure 2A). These findings were confirmed by coronary angiography in 2022. Similarly, both the Achilles tendon thickness (ATT) on ultrasonography and carotid intima-media thickness (IMT) significantly decreased with lomitapide treatment. Moreover, the minor soft plaque at the orifice of the right carotid artery became calcified with no progression with lomitapide therapy.

Case 2 is a 33-year-old man with a remarkable history related to HoFH. His first symptoms were xanthomas on his ankles and xanthelasma which appeared at 3 years of age. However, he was not diagnosed with HoFH until 20 years of age. He has presented with exertional angina and angiographically extensive ostial coronary stenotic lesions and had undergone CABG accompanied by aortic root dilation at 20 years of age. One year later, he was referred to our lipid clinic for extremely high LDL-cholesterol levels ranging between 420 and 540 mg/dl. Xanthomas were apparent on his elbows, knees, and ankles. He was already prescribed rosuvastatin (40 mg/day) and ezetimibe treatment, and we introduced LDL-apheresis. He lived a 14-h drive from our center; therefore, he receive monthly apheresis. As his LDL-cholesterol levels were still >200 mg/dl post-apheresis (Mach et al., 2020), we introduced lomitapide in June 2014, when he was 25 years of age. At the initial dose of oral 5 mg/day, mild nausea was observed. With the up-titrated dose of 20 mg/day, his nausea did not increase; however, after 6 months, he declined to take his pills as he experienced an 8 kg weight loss. For the next 3 years, he continued monthly apheresis but no lomitapide. We re-introduced lomitapide in November 2017 due to persistent LDL-cholesterol levels >500 mg/dl. However, this time he was compliant with the low-fat diet and lomitapide. He did not experience any adverse symptoms or weight loss with an up-titrated dose to 40 mg/day. No alteration was observed in transaminase levels, and a recent hepatic fibroscan was normal. We observed an additional 27% LDL-cholesterol reduction for a 5 mg daily dose of lomitapide at the end of the first month, which increased to 49% at the end of 6 months at a dose of 20 mg daily. The patient has been taking the current dose of lomitapide (40 mg/day) for almost 5 years and concomitant apheresis (every 2–3 months). During the pandemic, his access to apheresis was disturbed for 1 year; however, he maintained his lomitapide therapy. He also received supplementation with omega 3-6-9 and vitamin E. However, he reported several times reasons other than adverse effects for not being adherent to lomitapide therapy. During the follow-up, his coronary lesions and aortic stenosis progressed, and he underwent aortic valve replacement (Benthal operation), carotid endarterectomy, and repeated CABG in 2021. Figure 2B depicts the comparison of 2014 and 2019^−ΔΔCT^ angiograms showing significant progression of calcifications in the aortic root.

### 2.3 Genetic results

According to the sequencing results, Case 1 was heterozygous for *LDLR* [c.664T>C, (p.Cys222Arg)], while Case 2 was homozygous for *LDLR* [c.1760dupG, (p.Ser587ArgfsTer16)]. No clinically significant mutation was found in the *APOB, PCSK9*, and *LDLRAP1* regions in both patients. Additionally, 16 different *MTTP* variants were detected in the two patients. Cases 1 and 2 shared 2 *MTTP* variants (rs3816873 and rs1061271). Case 2 also showed one mutation (c.*450A>G) with uncertain clinical significance in public databases, while Case 1 showed 13 mutations (rs11944749, rs17029189, rs11944752, rs17029213, rs17029215, rs2306985, rs2718684 rs34734558, rs41275719, rs7667001, rs881981, rs982424, and rs991811) in *MTTP*. Twelve of these 13 mutations were categorized as benign/likely benign in public databases; only *MTTP* c.3G>A (rs11944752) was associated with elevated plasma glucose, insulin (MAGIC Consortium HGVM 4589669), and cholesterol levels (Teslovich et al., 2010).

## 3 Discussion

We present two real-life cases of HoFH with completely different disease courses after the commencement of lomitapide therapy. Case 1 demonstrated that long-term effective LLT with low-dose lomitapide combined with apheresis safely prevented the progression of atheroma both in coronary and vascular territories and retarded the progression of aortic stenosis. However, Case 2 showed a completely different course, with significant progression of his coronary lesions and aortic involvement. The patient had to undergo re-operation despite similar baseline LDL-cholesterol levels to those in Case 1. The difference between these patients’ disease courses suggests the importance of adherence to therapy for the clinical success of therapy.

Lomitapide is a new-generation potent lipid-lowering agent that acts independently of LDL receptors. Lomitapide inhibits MTTP, a cellular protein that transports neutral lipids between membrane vesicles and acts as a chaperone for the synthesis of apoB-containing triglyceride-rich lipoproteins. Lomitapide has a critical role in the assembly and secretion of apoB-containing lipoproteins in the liver and intestines (Hussain et al., 2012). Thus, lomitapide, besides triglycerides, effectively reduces LDL-cholesterol levels in patients who are lacking or have mutant LDLR. Lomitapide has been approved by the Food and Drug Administration as a lipid-lowering agent for patients with HoFH as an adjunct to standard LLT and by the European Medicines Agency for patients treated with standard LLT with or without apheresis.

Clinical evidence indicates lomitapide as a potent LDL-receptor-independent agent that has made it possible for patients with HoFH to reach their LDL-cholesterol goals. The phase 3 open-label dose-escalation trial demonstrated that lomitapide at maximal-tolerated doses (5–60 mg/day) reduced LDL-cholesterol by 50% at 26 weeks when added to the standard of care including lipid apheresis in 29 patients with HoFH (Cuchel et al., 2013). Moreover, long-term lomitapide treatment (range 2.1–5.7 years) with a median daily dose of 40 mg reduced LDL-cholesterol levels by an average of 45.5% in 19 patients with HoFH (Blom et al., 2017). During the 246-week therapy, 14 (74%) patients achieved the LDL-cholesterol target of 100 mg/dl and 11 (58%) patients reached the target of 70 mg/dl on at least 1 occasion. We observed a 31% additional reduction in LDL-cholesterol after 1 month of very low-dose (5 mg/day) lomitapide and 49% at the end of 6 months of therapy for a dose of only 20 mg/day in Case 1. In Case 2, the LDL-cholesterol levels were reduced by an additional 27% and 49% for 5 and 20 mg daily doses of lomitapide at the end of 1 and 6 months, respectively. Due to the effective reductions in LDL-cholesterol levels, we decreased the frequency of the apheresis sessions to every 2 months. The LDL-cholesterol reductions achieved in our cases are consistent with those in an HoFH case series reporting >50% reductions for doses of 10–40 mg/day in real-life settings (Stefanutti et al., 2016), which likely led to clinically significant reductions in ASCVD. Consequently, Case 1, who showed a consistent reduction of LDL-cholesterol levels, showed no ASCVD events and no atheroma progression. However, Case 2 showed a different course, with atherosclerosis progression during the 5-year therapy. The difference in therapy adherence was likely the major explanation for the diverse disease courses after lomitapide therapy in our patients. Adherence to LLT favors the survival of patients with ASCVD (Dopheide et al., 2021). Case 1 was adherent since the first dose of therapy. However, in Case 2, the first attempt to use lomitapide was not successful as the patient declined its use due to weight loss; another major obstacle was non-compliance to the low-fat diet, which is a requirement for lomitapide use. After 3 years, we re-introduced the drug. While he was adherent to the low-fat diet, the patient had many excuses for his lack of therapy adherence, likely due to his impulsive behaviors. Barratt impulsivity Scale 11 (Tamam et al., 2013) demonstrated the high impulsivity of Case 2 and the low impulsivity of Case 1. Treatment adherence is inversely related to impulsive and compulsive behaviors (López-Torrecillas et al., 2021).

Hepato-steatosis and liver-enzyme elevation are well-known phenomena associated with lomitapide treatment. However, none of these were observed in either of our patients with the long-term use of lomitapide at doses lower than those used in clinical trials. The only adverse effect was the weight loss reported by Case 2. These findings suggest that most of the adverse events can be avoided by lowering the dosing of lomitapide, as assessed by the LOWER study, in which the long-term (5-year) follow-up of patients on lomitapide (10–20 mg/dl) showed significantly lower adverse event rates compared to the results of the Phase-3 study conducted with doses of 40–60 mg/dl (Underberg et al., 2020). Furthermore, the 20 mg daily dose of lomitapide effectively reduced LDL-cholesterol levels almost to the targets in Case 1 in the present study. Increasing the dose to 40 mg likely would have allowed us to stop apheresis; however, as the patient insisted on apheresis therapy, we continued with 20 mg lomitapide combined with bi-monthly apheresis for >7 years without adverse effects. Low-dose lomitapide plus conventional therapy (statin and ezetimibe) with a decreased apheresis frequency probably increased the adherence to long-term therapy besides efficient LDL-lowering. Similarly, the use of combination therapy was associated with a higher proportion and greater odds of achieving the therapeutic LDL goals of <70 mg/dl and <55 mg/dl,, particularly with the combination of five drugs in the HICC registry (Vallejo-Vaz et al., 2021).

An important aspect of Case 1 in this study is the stabilized course of the aortic atheroma. In patients with late apheresis initiation after 8–10 years of age, the progression of aortic atheroma to stenosis reportedly cannot be prevented even if the LDL goals are attained (Cuchel et al., 2014; Kayikcioglu et al., 2018; Kayikcioglu, 2021). However, in Case 1, after commencing lomitapide use, the aortic involvement of the valvular stenosis and aortic plaques remained stable. This is also contrary to our clinical experience that despite meeting the LDL goals, children with late-onset apheresis therapy (after 8 years of age) show a gradual progression of aortic valve sclerosis or mild stenosis to moderate and severe stenosis.

In addition, our experiences with both of these patients call attention to lipid apheresis as a treatment difficult to access during the pandemic. Thus, novel effective LDL-lowering agents such as oral lomitapide may be useful for patients with HoFH during lockdowns due to pandemics or any other disaster that could disrupt access to apheresis as a hospital-based therapy (Kayikcioglu et al., 2020). Moreover, apheresis cessation or decreased frequency with an effective LDL-lowering agent will improve the psychosocial wellbeing of patients. We previously documented that apheresis as a semi-invasive, time-consuming therapy leads to a decreased quality of life, increased risk of depression, and deterioration in mental status, which lead to high refusal and low adherence rates (Kayikcioglu et al., 2019; Tunçel et al., 2020) noticeably leading to undertreatment of patients with HoFH, consequently affecting ASCVD outcomes.

Although both patients were phenotypically HoFH with untreated LDL-cholesterol levels >500 mg/dl, xanthomas appearing at early ages, and parental clinical features of heterozygous FH, Case 1 was heterozygous and Case 2 was homozygous for *LDLR* mutations. In addition to standard genetic analysis of the four associated genes, this study is the first to study *MTTP* in patients with HoFH. Loss-of-function mutations within *MTTP* result in severe autosomal recessive diseases, causing ataxia, failure to thrive, steatorrhea, and muscle weakness known as abetalipoproteinemia (Hussain et al., 2012). However, gain-of-function mutations in *MTTP* are not yet well understood. We identified several variants in both patients that may alter lipid levels or affect the clinical efficacy and adverse reactions to lomitapide.

The cases presented have some limitations and strengths. The evaluation of only five genes affecting LDL-cholesterol levels might be a limitation as other genes may explain the severe FH phenotype of the patients, especially in Case 1. However, the evaluation of *MTTP* variants is an important strength as these are the first case presentations of HoFH with *MTTP* variants. Detailed long-term follow-up of the cases and evaluation of impulsivity are additional strengths that allow the evaluation of adherence, clinical course, and the associated factors. Moreover, Case 1 is the first reported case to show the cessation of aortic atheroma progression despite the late onset of apheresis therapy, suggesting the importance of effective LDL-cholesterol lowering in combination with lomitapide and apheresis. Moreover, the comparison of two HoFH patients with similar lipid profiles at baseline and who received the same therapy allowed the assessment of the impact of adherence to therapy.

In conclusion, long-term, low-dose lomitapide was an effective and well-tolerated adjunct to conventional LLT and lipid apheresis in real life. Adherence to lomitapide and a low-fat diet in addition to standard LLT with decreased apheresis frequency appeared to be associated with more effective and safer management of HoFH and prevented the progression of atheroma burden in both the coronaries and aorta and regression in CIMT and ATT. Further studies are needed to illuminate the role of gain-of-function mutations in *MTTP* on lipid levels and response to lomitapide in patients with HoFH.

## Data Availability

The datasets for this article are not publicly available due to concerns regarding participant/patient anonymity. Requests to access the datasets should be directed to the corresponding author.
